# Xq26.2 Contiguous Gene Deletion Involving *FRMD7* and *IGSF1*: Highly Penetrant Infantile Nystagmus with Variable Endocrine Involvement

**DOI:** 10.3390/genes17080962

**Published:** 2026-08-17

**Authors:** Tomer Poleg, Lior Carmon, Elad Brav, Noam Hadar, Vadim Dolgin, Shirly Amar, Ginat Narkis, Ohad S. Birk, Libe Gradstein

**Affiliations:** 1 Faculty of Health Sciences, Ben-Gurion University of the Negev, Beer Sheva 8410501, Israel; 2 Pediatric Endocrinology Unit, Soroka University Medical Center, Beer Sheva 8410501, Israel; 3 Genetics Institute, Soroka University Medical Center, Beer Sheva 8410501, Israel; 4 The Danek Gertner Institute of Human Genetics, Sheba Medical Center, Tel-Hashomer, Ramat Gan 5266202, Israel; 5 Department of Ophthalmology, Soroka Medical Center and Clalit Health Services, Beer Sheva 8410501, Israel

**Keywords:** *FRMD7*, *IGSF1*, *ARHGAP36*, *OR13H1*, *STK26*, *ENOX2*, central hypothyroidism, nystagmus, structural variants (SVs), contiguous gene deletion

## Abstract

**Background/Objectives:** Contiguous Xq26.2 deletions involving *FRMD7* and *IGSF1* are rare and incompletely characterized. *FRMD7* loss causes X-linked idiopathic infantile nystagmus (IIN), whereas *IGSF1* loss causes central hypothyroidism and other pituitary-related abnormalities. We aimed to define the clinical and molecular spectrum of an Xq26.2 contiguous gene deletion in a large Bedouin kindred with infantile nystagmus. **Methods:** We clinically evaluated ten family members (3 males and 7 females); genomic analyses included chromosomal microarrays, whole-exome and whole-genome sequencing, breakpoint PCR, and Sanger sequencing. **Results:** We identified a novel 1.44-Mb Xq26.1-q26.2 deletion, NC_000023.11:g.130756102_132199443del (GRCh38), encompassing complete loss of *FRMD7*, *IGSF1*, *ARHGAP36*, *OR13H1*, and *STK26* and partial deletion of the 5′ end of *ENOX2*. The deletion was molecularly confirmed in six family members. The kindred exhibited a broad phenotypic spectrum, from an asymptomatic confirmed female carrier to isolated nystagmus and combined ocular–endocrine manifestations. Nystagmus was highly penetrant, whereas endocrine abnormalities were less frequent and variable. Hypoprolactinemia occurred in one confirmed female carrier, and a confirmed hemizygous boy had low-normal free T4 without overt central hypothyroidism. The only relative with overt central hypothyroidism was not genotyped; therefore, co-segregation could not be established. **Conclusions:** These findings further delineate the clinical spectrum of Xq26.2 deletions, highlight marked intrafamilial variability, and support structural variant analysis in unexplained IIN and/or endocrine abnormalities. Longitudinal endocrine assessment of individuals with Xq26.2 deletions and at-risk relatives in affected families may enable early detection and treatment of hypothyroidism.

## 1. Introduction

The Xq26.2 locus contains several functionally distinct genes, most notably *FRMD7* and *IGSF1*. *FRMD7*-related IIN usually begins within the first six months of life and is characterized by horizontal, conjugate, gaze-dependent nystagmus; jerk, pendular, and periodic alternating waveforms may occur. Visual acuity is generally preserved, although refractive errors, strabismus, amblyopia, and anomalous head posture may coexist. Ocular structures and electrophysiologic studies are typically normal. Hemizygous males show high penetrance, whereas heterozygous females show incomplete and variable penetrance [1,2,3,4].

Loss-of-function variants in *IGSF1*, which is expressed in the anterior pituitary, cause *IGSF1* deficiency syndrome. Central hypothyroidism typically presents with low free thyroxine and a low or inappropriately normal thyroid-stimulating hormone concentration and may be missed by neonatal screening programs based primarily on TSH. Additional reported features include hypoprolactinemia, delayed pubertal testosterone rise despite normal or early testicular growth, adult macroorchidism, occasional transient growth hormone deficiency, and increased adiposity. Female carriers usually have a milder and less penetrant biochemical phenotype [5,6,7,8].

Contiguous deletions at Xq26.2 involving *FRMD7* and *IGSF1* are rare and incompletely characterized [9]. Although the association between congenital nystagmus and central hypothyroidism was first recognized decades ago [10], a molecular basis involving combined loss of *FRMD7* and *IGSF1* has been reported in only a small number of families [9,11].

Here, we describe, to our knowledge, the largest reported kindred with combined loss of *FRMD7* and *IGSF1*: a large Bedouin family from southern Israel harboring the 1.44-Mb Xq26.1-q26.2 deletion NC_000023.11:g.130756102_132199443del. By integrating detailed ophthalmic and endocrine phenotyping with genomic analysis, we aim to delineate the clinical spectrum, assess apparent ocular and endocrine penetrance, and compare the kindred with previously reported Xq26.1-q26.2 deletions.

## 2. Methods

**Clinical Phenotyping and Ophthalmic Assessment:** Seven individuals (I-2, II-2, II-9, III-1, III-2, III-3, and III-5) underwent direct comprehensive ophthalmic examination, including visual acuity, ocular alignment, refractive status, characterization of nystagmus and head posture, slit-lamp biomicroscopy, and dilated fundus examination (Table 1). Ophthalmic information for deceased individual II-4 was obtained from family reports and available records; individuals III-7 and III-9 did not undergo a detailed ophthalmic examination. Optical coherence tomography was performed in III-1 and III-5; full-field electroretinography and flash visual evoked potentials were recorded in III-2 and III-3; and brain MRI was available for III-2.

**Endocrine, Systemic, and Neurodevelopmental Assessment:** Thyroid laboratory data were available for six individuals (II-2, II-9, III-1, III-2, III-3, and III-7). The measured analytes included serum TSH, free T4, and free T3, depending on sample availability; serum prolactin was available only for II-2. Test dates, numerical results, units, and laboratory reference intervals are provided in Table 2. Other endocrine, systemic, and neurodevelopmental findings were obtained from direct clinical evaluation, medical records, and family history. Autism spectrum disorder was not assessed using a standardized diagnostic instrument; statements regarding autism therefore refer only to the absence of a documented diagnosis or reported clinical concern.

**Molecular Genetic Analysis:** Next-generation sequencing, including whole-exome sequencing (WES) and whole-genome sequencing (WGS), was performed in individual III-3. NGS data were analyzed using VARista [12] and Franklin (Genoox; https://franklin.genoox.com/clinical-db/home, version 93, accessed on 20 April 2026), and Integrative Gen, omics Viewer (IGV, version 2.19.x, accessed on 20 April 2026) was used for read-level analysis. Breakpoint segregation analysis was conducted in individuals II-2, II-9, III-2, III-3, III-5, and III-9 using PCR followed by Sanger sequencing [13]. The primers used to amplify the deletion junction were forward 5′-TTTATCCTCAAAAGTTGTCAGACCT-3′ and reverse 5′-GCCCTGGGCTTAGTAGCTTG-3′. Chromosomal microarray analysis (CMA) was performed in individuals II-2, III-2, and III-3 and in one healthy control using the CytoScan™ 750K Array (Applied Biosystems™, Thermo Fisher Scientific Waltham, MA, USA), which contains 750,344 markers for copy-number analysis, including approximately 550,000 non-polymorphic markers and 200,000 SNP markers. Sample processing and hybridization followed the manufacturer’s protocol. Arrays were run on the GeneChip Fluidics Station 450 within the GeneChip System 3000, and data were analyzed using Chromosome Analysis Suite (ChAS) software (Thermo Fisher Scientific, Santa Clara, CA, USA).

## 3. Results

### 3.1. Clinical Findings

**Figure 1:**
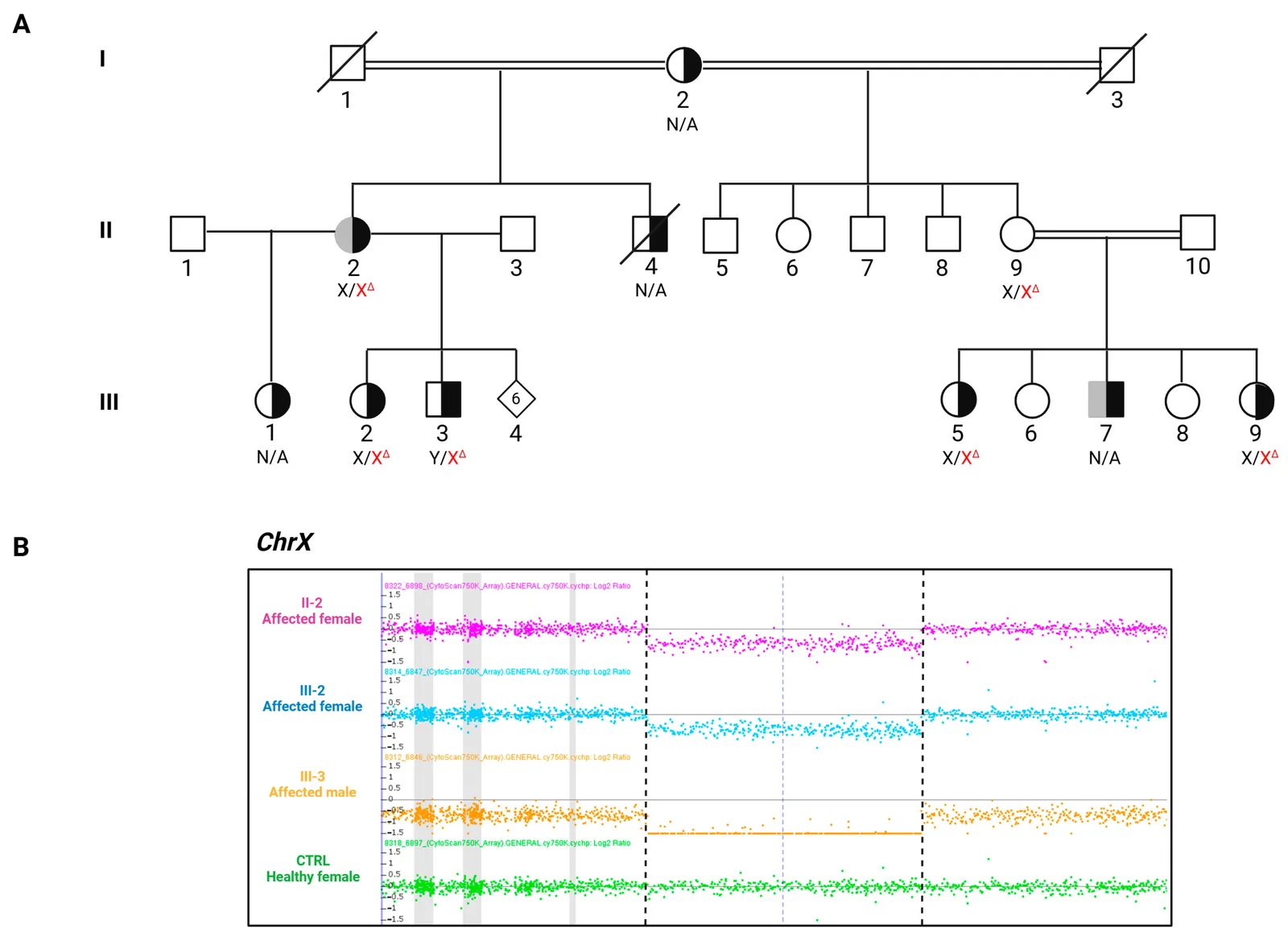
**Pedigree of the Bedouin kindred with Xq26.2 contiguous gene deletion.** (**A**) Pedigree of the studied family across three generations. Squares represent males, circles represent females and the diamond symbol represents siblings of unspecified sex (with the internal number ‘6’ indicating six individuals); diagonal lines indicate deceased individuals. Double horizontal mating lines indicate consanguineous unions. The two partners shown for individual I-2 reflect widowhood followed by remarriage, whereas the two partners shown for individual II-2 reflect divorce followed by remarriage. Filled black symbols denote individuals with nystagmus, while gray shading indicates endocrine abnormalities (hypoprolactinemia and/or central hypothyroidism). Half-filled symbols represent individuals exhibiting both ocular and endocrine phenotypes. The nystagmus status of deceased individual II-4 was based on family report. Genotypes are indicated where available ($X/X^{\Delta }$, $Y/X^{\Delta }$), **with red font (**$X^{\Delta }$**) highlighting the deletion allele**, and “N/A” denotes individuals for whom genetic testing was not performed. (**B**) Chromosomal Microarray (CMA) Analysis of Chromosome X. Log2 ratio plots from CMA using the CytoScan 750K Array. The x-axis represents the genomic position along the X chromosome, and the y-axis represents the Log2 ratio values. II-2 (magenta): affected female. III-2 (blue): affected female. III-3 (orange): affected male. CTRL (green): healthy control female. The gray stripes delineate International Standards for Cytogenomic Arrays (ISCA) CytoRegions. The dashed black lines indicate the boundaries of the detected copy number variation region.

We included ten members of a large Bedouin kindred (7 females and 3 males; ages 6–64 years). Six were molecularly confirmed deletion carriers (II-2, II-9, III-2, III-3, III-5, and III-9), whereas four clinically included relatives were not genotyped (I-2, II-4, III-1, and III-7). CMA and/or deletion-junction PCR followed by Sanger sequencing were used for molecular confirmation, as detailed for each individual in Table 1. Phenotypes ranged from an asymptomatic confirmed female carrier to isolated IIN. Individual III-7 had nystagmus and documented central hypothyroidism, but his relationship to the familial deletion could not be molecularly confirmed (Figure 1A; Table 1 and Table 2).

Seven individuals underwent direct ophthalmic examination, while ocular information for II-4, III-7, and III-9 was limited to records or family report. Nystagmus was reported in nine of ten family members and was absent only in confirmed female carrier II-9. In examined individuals, the nystagmus was horizontal and jerk-like and was generally more pronounced in lateral gaze. Visual acuity ranged from 6/6 to 6/21. Two individuals had an anomalous head posture, one had associated head shaking, two had esotropia, and one had amblyopia. Refractive errors included mild-to-moderate hypermetropia and mild-to-high astigmatism; mild, clinically insignificant lenticular opacities were observed in two individuals. Fundus examinations were normal in all seven directly examined individuals. OCT showed normal macular structure and foveal contour in III-1 and III-5; ffERG and FVEP responses were normal in III-2 and III-3; and brain MRI was normal in III-2.

Thyroid laboratory data were available for six individuals (II-2, II-9, III-1, III-2, III-3, and III-7). The measured analytes, test dates, numerical values, units, and laboratory reference intervals are presented in Table 2. Five of these individuals had no documented overt central hypothyroidism. Individual III-7 was diagnosed with central hypothyroidism at age 8 years and also had developmental delay. Despite attempts to recruit him, molecular testing could not be performed because of his significant intellectual disability, limited ability to cooperate with study procedures, and unwillingness to attend an additional evaluation; his carrier status therefore remains unconfirmed. Individual III-3, a confirmed hemizygous male aged 6 years, had a history of neonatal jaundice and a low-normal free T4 value (0.9 ng/dL; reference interval 0.8–1.5) with a normal TSH and did not meet biochemical criteria for overt central hypothyroidism. Prolactin was measured only in II-2 and was low (2 ng/mL; reference interval 3–29). Obesity was documented in three individuals.

Additional systemic findings, whose possible causation by the deletion is completely unclear, included congenital heart defects, Crohn’s disease, anxiety, diabetes or prediabetes, dyslipidemia, and breast cancer (Table 2).

No participant had a documented diagnosis of autism spectrum disorder. Because no standardized autism or neurodevelopmental instrument was administered, this observation should be interpreted as absence of a recorded diagnosis rather than formal exclusion of autism.

### 3.2. Identification of a Novel Large Deletion Within Chromosome X

**Figure 2:**
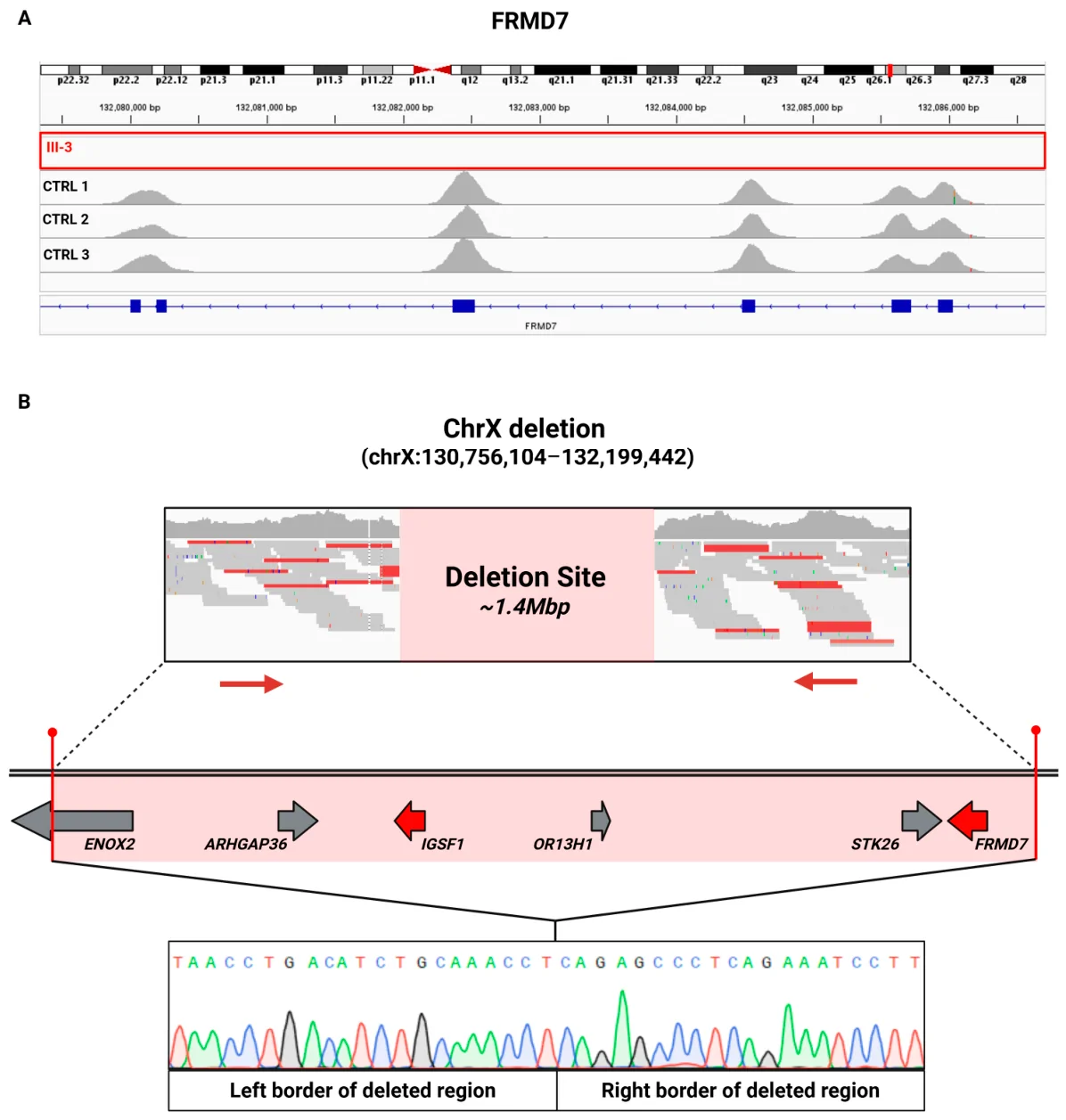
**Mapping and validation of the 1.44-Mb Xq26.2 deletion.** (**A**) IGV visualization of whole-exome sequencing data for individual III-3 and three unrelated controls, showing absent coverage across the *FRMD7* region in III-3. The red vertical line highlights the Xq26.2 deletion site, dark red triangles represent the centromere, and alternating black, gray, and white bands represent standard chromosome cytobands. (**B**) Whole-genome sequencing in III-3 identified discordant and clipped reads defining breakpoints at chrX:130,756,102 and chrX:132,199,443 (GRCh38), corresponding to NC_000023.11:g.130756102_132199443del. The schematic depicts complete deletion of *ARHGAP36*, *IGSF1*, *OR13H1*, *STK26*, and *FRMD7* and partial deletion of *ENOX2*. The Sanger chromatogram shows the deletion-junction sequence.

Affected individuals underwent standard clinical genetic evaluation, including karyotyping and targeted gene panel testing, which yielded negative results. CMA was subsequently performed in II-2, III-2, and III-3 and demonstrated consistent log2 ratio deviations at Xq26.2, indicating the familial copy-number loss. The log2 ratio shift was more pronounced in hemizygous male III-3, as expected for an X-chromosomal deletion (Figure 1B). WES and WGS were performed in III-3. WES revealed no pathogenic single-nucleotide or small insertion/deletion variants in known or candidate disease-associated genes, while IGV visualization confirmed absence of aligned reads across *FRMD7* compared with controls (Figure 2A).

Whole-genome sequencing in III-3 identified clipped and discordant reads at chrX:130,756,102 and chrX:132,199,443 (GRCh38), defining a 1,443,342-bp deletion, NC_000023.11:g.130756102_132199443del (Figure 2B). Read-depth analysis showed complete loss of coverage across the interval. The deletion completely encompasses *ARHGAP36*, *IGSF1*, *OR13H1*, *STK26*, and *FRMD7*. The proximal breakpoint lies within *ENOX2* transcript NM_006375.4; approximately 147 kb of the 5′ portion of *ENOX2*, including three of its 15 coding exons, is deleted and is predicted to cause loss of function of the affected allele.

Breakpoint PCR followed by Sanger sequencing confirmed the deletion junction in six family members (II-2, II-9, III-2, III-3, III-5, and III-9), whereas the four clinically included relatives I-2, II-4, III-1, and III-7 were not molecularly tested. The structural variant was submitted to ClinVar under submission identifier SUB16377080; a public ClinVar accession number was not yet available at the time of revision.

## 4. Discussion

In 1969, Schulman and Crawford described a boy with congenital nystagmus and central hypothyroidism [10]. Two subsequent reports identified Xq26.1-q26.2 deletions encompassing both *FRMD7* and *IGSF1* [9,11] in single patients: Reynaert et al. described a male newborn with a ~1.29 Mb deletion, bilateral nystagmus, and central hypothyroidism; hypoprolactinemia and testicular enlargement were not present at that early age [9]. AlMoallem et al. reported a 14-year-old male with a similar deletion, early-onset nystagmus, autism spectrum disorder, and global developmental delay. The deletion was inherited from a mildly affected mother, and no thyroid or pubertal abnormalities were reported; the authors considered the cause of the neurodevelopmental phenotype uncertain [11].

To our knowledge, the present kindred is the largest family reported to date with combined loss of *FRMD7* and *IGSF1*. The deletion was molecularly confirmed in six family members, whereas four clinically included relatives were untested. Nystagmus was highly penetrant among confirmed carriers and clinically affected relatives, whereas endocrine findings were less frequent and incompletely ascertained. Confirmed hemizygous male III-3 had nystagmus without overt central hypothyroidism. Individual III-7 had both nystagmus and central hypothyroidism, but because he was not genotyped, co-segregation of the overt thyroid phenotype with the familial deletion cannot be established. Among females, confirmed carrier II-2 had nystagmus and hypoprolactinemia, whereas confirmed carrier II-9 was clinically asymptomatic.

Central hypothyroidism is highly penetrant in hemizygous males with pathogenic *IGSF1* variants; in the largest published clinical series, all evaluated males had central hypothyroidism [6,8]. However, hemizygous male relatives with free T4 concentrations just above the lower limit of the reference interval have also been reported [14]. Consequently, the absence of overt central hypothyroidism in confirmed hemizygous male III-3 at age 6 years represents milder-than-expected or borderline thyroid involvement rather than proven non-penetrance. His neonatal jaundice and low-normal free T4 value do not establish central hypothyroidism. Possible explanations include his young age, limited longitudinal biochemical sampling, age- and assay-dependent reference intervals, incomplete evaluation of other pituitary axes, or genuine variable expression. Longitudinal assessment of free T4 and TSH, growth, and pubertal development is therefore appropriate; treatment decisions should be based on established clinical and biochemical criteria. The central hypothyroidism in III-7 is clinically compatible with *IGSF1* deficiency, but its causal relationship to the deletion and its contribution to his intellectual disability remain uncertain because he was not genotyped.

Female *IGSF1* carriers generally have milder biochemical findings. In a large series, 18% had low free T4, 60% had low-normal free T4, and 22% had mild prolactin deficiency [8]. These data are consistent with hypoprolactinemia in confirmed carrier II-2 and the absence of overt central hypothyroidism in the tested females in our kindred. Obesity was recorded in three female family members, although the incomplete metabolic and endocrine assessment precludes attributing this finding to *IGSF1* deficiency.

The ocular variability in this pedigree, including an asymptomatic confirmed female carrier, is consistent with *FRMD7*-associated IIN. A 2026 analysis combining a large in-house cohort with published cases reported penetrance of 100% in males and 39.3% in females, supporting high male penetrance and incomplete, variable expression in heterozygous females [4]. Skewed X-chromosome inactivation has been proposed as a modifier, but published studies have not demonstrated a consistent relationship between blood X-inactivation patterns and the ocular phenotype; additional genetic, epigenetic, or environmental modifiers may contribute [1,3,4].

Table 3 compares the present kindred with the two previously reported Xq26.1-q26.2 deletions encompassing both *FRMD7* and *IGSF1* and with the 2025 *IGSF1*-containing deletion that spared *FRMD7*. The two combined-gene deletions showed differing endocrine and neurodevelopmental findings [9,11]. Tolmacheva et al. reported two brothers with a smaller deletion involving *IGSF1* but sparing *FRMD7*; both had mild congenital hypothyroidism, obesity, enlarged testes, and neurodevelopmental and behavioral abnormalities, while nystagmus was not reported [15]. This comparison supports separation of the ocular phenotype from the endocrine phenotype and illustrates that neurodevelopmental abnormalities are not specific to combined loss of *FRMD7* and *IGSF1*.

The deleted interval we report comprises complete deletion of five genes (*ARHGAP36*, *IGSF1*, *OR13H1*, *STK26*, and *FRMD7*) and disruption of *ENOX2*. The proximal breakpoint lies within the *ENOX2* transcript NM_006375.4, deleting approximately 147 kb of the 5′ portion of the gene, including three of 15 coding exons; this is predicted to cause loss of function of the affected allele. The clinical relevance of *ARHGAP36*, *OR13H1*, *STK26*, and *ENOX2* remains uncertain. According to the Human Gene Mutation Database [16], none has been definitively associated with a monogenic human disorder. The ocular and endocrine findings are therefore most plausibly related to loss of *FRMD7* and *IGSF1*, respectively, although a contribution from the wider deletion cannot be excluded.

Our data do not permit a definitive conclusion regarding autism or broader neurodevelopmental effects. No participant had a documented autism diagnosis, but standardized assessment was not performed. In the AlMoallem report, the proband with autism and global developmental delay also carried a 7q22.1 duplication, and the authors considered causality uncertain [11]. The brothers reported by Tolmacheva had neurodevelopmental and behavioral abnormalities with an *IGSF1*-containing deletion that spared *FRMD7* [15]. In our kindred, III-7 had intellectual disability but was not genotyped, and the mild attention difficulties in III-3 are nonspecific. Thus, neurodevelopmental findings cannot currently be attributed specifically to combined *FRMD7*/*IGSF1* deletion.

Clinically, the combination of infantile nystagmus and central hypothyroidism should prompt consideration of an Xq26.2 deletion involving *FRMD7* and *IGSF1*. In children with unexplained IIN, particularly males with developmental concerns, prolonged neonatal jaundice, abnormal growth or pubertal development, or other pituitary features, thyroid evaluation should include both free T4 and TSH because TSH-based screening alone can miss central hypothyroidism. Individuals with a molecularly confirmed deletion involving *IGSF1* should receive longitudinal endocrine follow-up through childhood and puberty, with assessment of thyroid function, growth, prolactin, and pubertal development as clinically indicated [7,8].

From a diagnostic perspective, this study demonstrates complementary roles for CMA, WES/WGS read-depth and breakpoint analysis, and deletion-junction PCR/Sanger sequencing. These findings highlight the importance of structural-variant analysis in unresolved IIN and central hypothyroidism.

## 5. Limitations

This study has several limitations. Only six of the ten included family members were molecularly confirmed to carry the deletion. Most importantly, III-7, the only individual with documented overt central hypothyroidism, could not undergo molecular testing because of significant intellectual disability and limited cooperation and did not agree to attend an additional evaluation; his carrier status therefore remains unconfirmed. Direct comprehensive ophthalmic examinations were available for seven individuals, thyroid laboratory data for six, and prolactin for only one. Data for several relatives were obtained retrospectively from records or family reports. Growth hormone/IGF-1 status, gonadal function, pubertal development, and testicular volume were not systematically assessed, and III-3 is still too young for adult *IGSF1*-related features such as macroorchidism to be evaluated. Autism was not assessed with a standardized instrument. These incomplete assessments limit estimates of endocrine and neurodevelopmental penetrance, while the young age of some participants limits evaluation of later-onset endocrine manifestations, particularly pubertal abnormalities and macroorchidism. Finally, no functional studies were performed.

## 6. Conclusions

We describe, to our knowledge, the largest kindred reported to date with a molecularly defined 1.44-Mb Xq26.2 deletion causing complete loss of *FRMD7*, *IGSF1*, *ARHGAP36*, *OR13H1*, and *STK26* and partial loss of *ENOX2*. The kindred exhibited a broad phenotypic spectrum, from an asymptomatic confirmed female carrier to isolated nystagmus and ocular–endocrine manifestations within the kindred. Nystagmus was highly penetrant, whereas endocrine abnormalities were less frequent and variable. These findings further delineate the clinical spectrum of Xq26.2 deletions, highlight marked intrafamilial variability, and support structural variant analysis in patients with unexplained IIN and/or endocrine abnormalities. Longitudinal endocrine assessment of individuals with Xq26.2 deletions and at-risk relatives in affected families may facilitate early detection and treatment of hypothyroidism and other endocrine manifestations.

## Figures and Tables

**Table 1:** **Ophthalmic findings and molecular confirmation in members of the studied kindred.** Direct comprehensive ophthalmic examinations were performed in I-2, II-2, II-9, III-1, III-2, III-3, and III-5. Findings for II-4 were based on family report and available records; III-7 and III-9 did not undergo detailed ophthalmic examination. CMA, chromosomal microarray analysis; ffERG, full-field electroretinography; FVEP, flash visual evoked potentials; NA, not assessed; NLD, nasolacrimal duct; OCT, optical coherence tomography; OD, right eye; OS, left eye; OU, both eyes; PCR, polymerase chain reaction; VA, visual acuity; WES, whole-exome sequencing; WGS, whole-genome sequencing.

Case	Age/Sex	Genetic Testing/Molecular Confirmation	Nystagmus	Head Posture	Visual Acuity	Strabismus/Amblyopia	Refractive Error	Lens	Fundus	Other Ophthalmic Findings
**I-2**	64/F	Not tested (clinical only)	Yes	NA	OD 6/15, OS 6/10	No	NA	Clear	Normal	NA
**II-2**	36/F	CMA; Sanger	Yes	NA	OD 6/9, OS 6/7.5	No	OD +0.50 −0.75 × 25; OS +0.25	Clear	Normal	NA
**II-4**	20/M at death	Not tested (family report)	Yes	NA	NA	NA	Wore spectacles; detailed refraction unavailable.	NA	NA	Ocular history based on family report.
**II-9**	46/F	Sanger	No	NA	OD 6/6, OS 6/6	No	NA	Clear	Normal	NLD obstruction managed surgically.
**III-1**	16/F	Not tested (clinical only)	Yes	Head shaking; mild head turn to the right	OD 6/12, OS 6/12	No	OD +1.25 −1.00 × 10; OS +1.75 −1.75 × 170	Fine opacity OD	Normal	OCT: normal macular structure and foveal contour OU.
**III-2**	7/F	CMA; Sanger	Yes	NA	OD 6/7.5, OS 6/7.5	Esotropia	OD +2.50 −2.00 × 180; OS +2.25 −1.25 × 180	Clear	Normal	Brain MRI normal; ffERG and FVEP normal.
**III-3**	6/M	CMA; WES; WGS; Sanger	Yes	Head turn to the left; chin down	OD 6/15, OS 6/21	Esotropia; amblyopia OS	OD +4.50 −1.25 × 180; OS +4.50 −1.25 × 180	Clear	Normal	ffERG and FVEP normal.
**III-5**	19/F	Sanger	Yes	NA	OD 6/21, OS 6/18	No	OD +2.25 −3.75 × 180; OS +2.50 −4.00 × 10	Fine opacities OU	Normal	OCT: normal macular structure and foveal contour OU.
**III-7**	29/M	Not tested (clinical only)	Yes	NA	NA	NA	NA	NA	NA	NA
**III-9**	27/F	Sanger	Yes	NA	NA	NA	NA	NA	NA	NA

**Table 2:** **Endocrine laboratory results and selected systemic findings in members of the studied kindred.** Reference ranges are shown in parentheses. FT3, free triiodothyronine; FT4, free thyroxine; NA, not assessed or not available; T2DM, type 2 diabetes mellitus; TSH, thyroid-stimulating hormone; VSD, ventricular septal defect.

Case	Age/Sex	Test Date(s)	Free T4	Free T3	TSH	Prolactin	Other Endocrine/Systemic Findings
I-2	64/F	NA	NA	NA	NA	NA	Obesity; T2DM; dyslipidemia; breast cancer.
II-2	36/F	28 October 2025	13.8 pmol/L (11.5–22.7)	4.4 pmol/L (3.5–6.5)	0.26 µIU/mL (0.55–4)	2 ng/mL (3–29)	None
II-4	20/M at death	NA	NA	NA	NA	NA	No endocrine data; died in a motor vehicle accident at age 20.
II-9	46/F	15 June 2023	1.0 ng/dL (0.8–1.5)	3.5 pg/mL (2.3–4.2)	1.579 µIU/mL (0.4–4)	NA	Prediabetes; anxiety; bicuspid aortic valve.
III-1	16/F	22 March 2023	1.2 ng/dL (0.8–1.5)	2.9 pg/mL (2.3–4.2)	1.779 µIU/mL (0.4–4)	NA	Obesity.
III-2	7/F	20 June 2023	1.3 ng/dL (0.8–1.5)	5.3 pg/mL (2.3–4.2)	1.376 µIU/mL (0.4–4)	NA	None
III-3	6/M	20 June 2023	0.9 ng/dL (0.8–1.5)	3.6 pg/mL (2.3–4.2)	0.773 µIU/mL (0.4–4)	NA	Neonatal jaundice; VSD; pulmonary stenosis; mild attention difficulties.
III-5	19/F	NA	NA	NA	NA	NA	Crohn’s disease.
III-7	29/M	23 May 2004	0.6 ng/dL (0.8–1.5)	NA	6.34 µIU/mL (0.4–4)	NA	Central hypothyroidism diagnosed at age 8 years; intellectual disability.
		5 August 2004	0.7 ng/dL (0.8–1.5)	3.2 pg/mL (2.3–4.2)	<0.05 µIU/mL (0.4–4)		
		20 November 2008	0.8 ng/dL (0.8–1.5)	NA	3.78 µIU/mL (0.4–4)		
		1 December 2016	NA	NA	2.16 µIU/mL (0.4–4)		
III-9	27/F	NA	NA	NA	NA	NA	Obesity.

**Table 3:** Comparison of reported Xq26.1-q26.2 deletions involving *IGSF1*, with or without *FRMD7*.

Report and Participants	Deletion	Ocular Phenotype	Endocrine, Neurodevelopmental, and Other Findings
Reynaert et al. (2015) [9]; one male newborn	1.29 Mb, Xq26.1-q26.2; arr Xq26.1q26.2(129,928,356–131,292,675)x0 (genome build not stated). Deleted genes included *FRMD7*, *IGSF1*, *ENOX2*, *ARHGAP36*, *OR13H1*, *FIRRE*, and *STK26/MST4*.	Bilateral nystagmus in the neonatal period.	Central hypothyroidism detected on day 3 (free T4 0.52 ng/dL; TSH 10.6 mU/L). Prolactin was elevated and testicular volume was normal at that age.
AlMoallem et al. (2015) [11]; 14-year-old male and mildly affected female relatives	1.29 Mb, Xq26.1-q26.2; arr Xq26.1q26.2(129,928,356–131,292,675)x0 (genome build not stated). Deleted genes included *FRMD7*, *IGSF1*, *ENOX2*, *ARHGAP36*, *OR13H1*, *FIRRE*, and *STK26/MST4*. A 370-kb 7q22.1 duplication was also present in the proband.	Nystagmus noted from age 1.5 years; the mother, maternal aunt, and a female cousin were mildly affected.	No thyroid or pubertal abnormality was reported. The proband had autism spectrum disorder and global developmental delay; the contribution of the X-chromosomal deletion was considered uncertain.
Tolmacheva et al. (2025) [15]; two brothers aged 2 and 8 years	1.16 Mb, arr[GRCh38] Xq26.1q26.2(130,662,822–131,826,589)x0. The deletion included *IGSF1*, *ENOX2*, *ARHGAP36*, *OR13H1*, *FIRRE*, *LOC105373338*, and *LINC01201*; *FRMD7* was spared.	Nystagmus was not reported.	Mild congenital hypothyroidism, obesity, enlarged testes, developmental and speech delay, aggressive behavior, emotional instability, and hepatomegaly.
Present kindred; ten family members aged 6–64 years	1.44 Mb; arr[GRCh38] Xq26.1q26.2(130756102_132199443)x0; NC_000023.11:g.130756102_132199443del. Complete deletion of *FRMD7*, *IGSF1*, *ARHGAP36*, *OR13H1*, and *STK26*, with deletion of approximately 147 kb of the 5′ portion of *ENOX2* (NM_006375.4), including three of 15 coding exons. Six individuals were molecularly confirmed; four were clinically included but untested.	Nystagmus reported in 9/10; horizontal jerk nystagmus, usually gaze-dependent. Visual acuity 6/6–6/21; variable anomalous head posture, refractive error, esotropia, and amblyopia. Retinal and optic nerve investigations were normal where performed.	Confirmed female carrier II-2 had hypoprolactinemia. Confirmed male III-3 had low-normal free T4 without overt central hypothyroidism at age 6. III-7 had overt central hypothyroidism and developmental delay but was not genotyped. No documented autism diagnosis; standardized assessment was not performed.

Abbreviations: TSH, thyroid-stimulating hormone. Coordinates and genome builds are reproduced as reported in the source publications; for the present kindred, coordinates correspond to the interval shown in Figure 2.

## Data Availability

The Xq26.2 deletion was submitted to ClinVar under submission identifier SUB16377080; a public accession number was pending at the time of revision. Additional data supporting the findings are available from the corresponding authors upon reasonable request.

## References

[1] Watkins R.J., Thomas M.G., Talbot C.J., Gottlob I., Shackleton S. (2012). The role of FRMD7 in idiopathic infantile nystagmus. J. Ophthalmol..

[2] Tarpey P., Thomas S., Sarvananthan N., Mallya U., Lisgo S., Talbot C.J., Roberts E.O., Awan M., Surendran M., McLean R.J. (2006). Mutations in *FRMD7*, a newly identified member of the FERM family, cause X-linked idiopathic congenital nystagmus. Nat. Genet..

[3] Choi K.D., Choi J.H. (2020). FRMD7-associated infantile nystagmus syndrome. J. Interdiscip. Genom..

[4] Liu S., Li S., Wang Y., Sun W., Hejtmancik J.F., Zhang Q. (2026). Genetic landscape and clinical characterization of FRMD7-related infantile nystagmus based on large in-house datasets and literature review. Investig. Ophthalmol. Vis. Sci..

[5] Bernard D.J., Brûlé E., Smith C.L., Joustra S.D., Wit J.M. (2018). From consternation to revelation: Discovery of a role for IGSF1 in pituitary control of thyroid function. J. Endocr. Soc..

[6] Sun Y., Bak B., Schoenmakers N., van Trotsenburg A.S.P., Oostdijk W., Voshol P., Cambridge E., White J.K., le Tissier P., Gharavy S.N.M. (2012). Loss-of-function mutations in IGSF1 cause an X-linked syndrome of central hypothyroidism and testicular enlargement. Nat. Genet..

[7] Persani L., Bonomi M. (2017). The multiple genetic causes of central hypothyroidism. Best Pract. Res. Clin. Endocrinol. Metab..

[8] Joustra S.D., Heinen C.A., Schoenmakers N., Bonomi M., Ballieux B.E.P.B., Turgeon M.-O., Bernard D.J., Fliers E., van Trotsenburg A.S.P., Losekoot M. (2016). IGSF1 deficiency: Lessons from an extensive case series and recommendations for clinical management. J. Clin. Endocrinol. Metab..

[9] Reynaert N., Braat E., de Zegher F. (2015). Congenital nystagmus and central hypothyroidism. Int. J. Pediatr. Endocrinol..

[10] Schulman J.D., Crawford J.D. (1969). Congenital nystagmus and hypothyroidism. N. Engl. J. Med..

[11] AlMoallem B., Bauwens M., Walraedt S., Delbeke P., De Zaeytijd J., Kestelyn P., Meire F., Janssens S., Van Cauwenbergh C., Verdin H. (2015). Novel *FRMD7* mutations and genomic rearrangement expand the molecular pathogenesis of X-linked idiopathic infantile nystagmus. Investig. Ophthalmol. Vis. Sci..

[12] Hadar N., Dolgin V., Oustinov K., Yogev Y., Poleg T., Safran A., Freund O., Agam N., Jean M.M., Proskorovski-Ohayon R. (2024). VARista: A free web platform for streamlined whole-genome variant analysis across T2T, hg38, and hg19. Hum. Genet..

[13] Poleg T., Hadar N., Kristal E., Roberts N.Y., Dolgin V., Aminov I., Safran A., Agam N., Jean M., Freund O. (2025). Early-onset movement disorder syndrome caused by biallelic variants in PDE1B encoding phosphodiesterase 1B. Mov. Disord..

[14] Ghanny S., Zidell A., Pedro H., Joustra S.D., Losekoot M., Wit J.M., Aisenberg J. (2021). The IGSF1 deficiency syndrome may present with normal free T4 levels, severe obesity, or premature testicular growth. J. Clin. Res. Pediatr. Endocrinol..

[15] Tolmacheva E.N., Kashevarova A.A., Fonova E.A., Salyukova O.A., Seitova G.N., Nazarenko L.P., Agafonova A.A., Minaycheva L.I., Ravzhaeva E.G., Petrova V.V. (2025). Prevalence of CNVs on the X chromosome in patients with neurodevelopmental disorders. Mol. Cytogenet..

[16] Stenson P.D., Mort M., Ball E.V., Chapman M., Evans K., Azevedo L., Hayden M., Heywood S., Millar D.S., Phillips A.D. (2020). The Human Gene Mutation Database (HGMD®): Optimizing its use in a clinical diagnostic or research setting. Hum. Genet..

